# Effects of Legacy PFAS and Novel Alternatives on Thyroid Function in Mother‒Child Populations: A Systematic Review

**DOI:** 10.1155/jt/1578567

**Published:** 2026-05-13

**Authors:** Lemin Gong, Yuhan Zhou

**Affiliations:** ^1^ School of Exercise and Health, Shanghai University of Sport, Shanghai, 200438, China, sus.edu.cn

**Keywords:** legacy per- and polyfluoroalkyl substances, mechanism, mother‒child, novel alternative, thyroid function

## Abstract

Per‐ and polyfluoroalkyl substances (PFAS) are extensively utilized in industrial and consumer applications, such as firefighting foams, textiles, electronics, industrial coatings, food packaging, and household consumer products, due to their excellent chemical stability. However, legacy long‐chain PFAS are prohibited because of their endocrine‐disrupting properties, bioaccumulative nature, and potential carcinogenicity, followed by the emergence of new alternatives, such as short‐chain congeners and novel PFAS alternatives. PFAS are known to disrupt endocrine function, and the thyroid gland, a critical endocrine organ, is particularly vulnerable to their effects. Mother‒child populations are more likely to be affected by PFAS due to their specific physiological characteristics, metabolic differences, and developmental stages. This paper reviews the exposure levels of legacy PFAS and their alternatives in maternal and infant blood and breast milk, and the relationship between PFAS exposure and thyroid hormone (TH) levels. It also examines the similarities and differences in the mechanisms by which different PFAS affect thyroid function. Research has revealed that both legacy and PFAS alternatives have been detected in maternal blood, infant blood, and breast milk. While legacy PFAS exhibit higher exposure levels with notable regional variations, the concentrations of PFAS alternatives, although lower, remain a concern. PFAS exposure affects TH in mothers and infants, with legacy PFAS having a more significant effect. The mechanisms underlying thyroid disruption caused by different PFAS are largely similar. However, PFAS alternatives are characterized by high protein affinity, ability to disrupt fatty acid metabolism, and high hydrophilicity, and may also increase the risk of thyroid cancer (TC). This study provides critical evidence for evaluating the safety of PFAS alternatives and provides a scientific foundation for developing preventive and regulatory measures.

## 1. Introduction

Per‐ and polyfluoroalkyl substances (PFAS) are a group of synthetic compounds that are extensively manufactured and utilized in outdoor gear, furniture, nonstick pans, electronics, fabrics, and cosmetics [1], due to their excellent surfactant properties, thermal stability, and chemical resistance [2]. However, long‐chain PFAS, such as perfluorooctane sulfonate (PFOS) and perfluorooctanoic acid (PFOA), are restricted by their persistence in the environment, bioaccumulation in organisms, endocrine‐disrupting properties, and potential carcinogenicity [3, 4]. Consequently, short‐chain alternatives, such as perfluorohexane sulfonate (PFHxS) and perfluorobutane sulfonate (PFBS), as well as emerging PFAS like hexafluoropropylene oxide dimer acid (HFPO‐DA, Gen‐X) and chlorinated polyfluoroalkyl ether sulfonate (Cl‐PFESA) have been introduced [5, 6]. Despite their widespread use, current research on these PFAS alternatives is fraught with gaps. Preliminary evidence suggests that these alternatives may exhibit greater cytotoxicity, but their environmental behavior, bioaccumulation potential, and long‐term health impacts, particularly their carcinogenicity and endocrine‐disrupting properties, are poorly understood [7, 8]. Thus, further in‐depth research into their safety is urgently needed.

While genetic predisposition, radiation exposure, metabolic syndrome (including obesity, hypertension, hyperlipidemia, and diabetes), and unhealthy lifestyle factors are recognized contributors, increasing evidence suggests that long‐term exposure to environmental pollutants, including PFAS, may also increase the risk of thyroid cancer (TC) [9, 10]. PFAS are classified as endocrine‐disrupting chemicals (EDCs) [4], and the thyroid, a critical endocrine organ, is particularly vulnerable to their effects [11]. Recent epidemiological studies have revealed positive associations between environmental EDCs and an increased incidence of thyroid disorders [12, 13].

Mother‒child populations face unique risks from PFAS exposure due to their distinct physiological characteristics, metabolic differences, and developmental stages. For pregnant and lactating women, primary exposure pathways include consumption of protein‐rich foods (e.g., meat, eggs, and dairy) and drinking water contaminated with PFAS, direct contact with PFAS‐containing consumer products (e.g., food packaging, textiles, and household cleaners), and inhalation or ingestion of environmental media (e.g., air, soil, and dust) [14–16]. PFAS can efficiently cross the placental barrier to reach the developing fetus, while infants are further exposed through breastfeeding and postnatal contact with PFAS‐contaminated environments [17–19]. Therefore, identifying and mitigating PFAS exposure in mother‒child populations is of paramount importance.

This study synthesizes existing research to examine PFAS levels in maternal and infant blood and breast milk, analyzes the association between PFAS exposure and thyroid hormone (TH) levels, and explores the mechanisms by which both legacy and alternative PFAS affect thyroid function. By highlighting the similarities and differences in their effects, this review aims to guide the formulation of targeted prevention and control measures. Notably, a recent systematic review and meta‐analysis by Du et al. summarized pooled evidence on PFAS exposure and thyroid health across the general population, including pregnant women [20]. The present review further extends this evidence framework by specifically focusing on mother–child pairs, comparing legacy PFAS and novel alternatives, and clarifying their distinct exposure profiles, placental transfer, and thyroid‐disrupting mechanisms, thus providing more targeted insights for this vulnerable population.

## 2. Short‐Chain Alternatives and Novel Alternatives for PFOA and PFOS

With increasing restrictions on the use of PFOA and PFOS due to their environmental persistence and potential adverse effects on human health, research into their short‐chain alternatives and novel alternatives has gained significant attention. These alternatives are designed to mitigate the environmental and health risks associated with legacy PFAS while retaining their functional properties. However, emerging studies have revealed that these alternatives may exhibit similar biotoxicity, raising concerns about their safety and underscoring the need for stringent regulatory measures [21].

### 2.1. Short‐Chain Alternatives and Novel Alternatives for PFOA

The short‐chain alternatives of PFOA primarily include perfluorobutanoic acid (PFBA), perfluoroheptanoic acid (PFHpA), perfluorohexanoic acid (PFHxA), and perfluoropentanoic acid (PFPeA) [22]. These compounds, characterized by their lower molecular weights, exhibit distinct environmental behaviors and toxicological profiles compared with long‐chain alternatives. Despite these differences, their widespread use has raised significant concerns, as emerging studies have detected their presence in various environmental media, indicating that they may still pose potential ecological and health risks [23].

Among the novel alternatives for PFOA, GenX and ammonium 4,8‐dioxa‐3H‐perfluorononanoate (ADONA) are two prominent alternatives [20]. Research indicates that placental trophoblast cells may serve as direct targets of GenX exposure, with studies demonstrating that GenX can disrupt gene expression and cellular function in trophoblast cells at concentrations below the cytotoxic threshold [24]. While ADONA is currently detected at minimal levels in the environment, it has been identified in surface water samples across the globe, raising concerns about its potential for widespread environmental distribution [23].

### 2.2. Short‐Chain Alternatives and Novel Alternatives for PFOS

Short‐chain alternatives of PFOS, such as PFBS and PFHxS, have been frequently detected in environmental samples [21]. Research has indicated that PFHxS can accumulate in humans, especially in firefighters, due to their occupational exposure to firefighting foams containing this compound [25].

Among the novel alternatives for PFOS, 6:2 fluorotelomer sulfonate (6:2 FTS) and Cl‐PFESA, especially 6:2 Cl‐PFESA, are notable for their persistence in humans. Among PFAS, 6:2 Cl‐PFESA has the longest median elimination half‐life (15.3 years), surpassing even PFOS (half‐life: 6.7 years) [26]. This suggests that, in terms of human bioaccumulation, 6:2 Cl‐PFESA may pose a greater concern than PFOS itself (see Table 1).

**TABLE 1:** Characteristics of traditional, short‐chain, and novel alternatives.

Category	Representative compounds	Carbon chain length	Environmental persistence	Bioaccumulation potential	Placental transfer efficiency	Key thyroid‐disrupting features
Legacy PFAS	PFOA, PFOS	Long (C8–C10)	High	High	Moderate	Interfere with TH synthesis, transport, and receptor signaling
Short‐chain alternatives	PFBA, PFPeA, PFHxA, PFHpA; PFBS, PFPeS, PFHxS	Short (C4–C7)	Low to moderate	Low to moderate	High	Strong protein‐binding affinity; disrupt TH transport
Novel alternatives	GenX, ADONA, FTS, Cl‐PFESA…	Variable (ether/modified structures)	Low to moderate	Low	High	High hydrophilicity; accumulates in thyroid tissue; links to fatty acid metabolism disturbance and elevated thyroid cancer risk

## 3. Materials and Methods

This systematic review was performed in accordance with the Preferred Reporting Items for Systematic Reviews and Meta‐Analyses (PRISMA) guidelines.

### 3.1. Search Strategy

Literature searches were conducted in electronic databases (PubMed, Web of Science, and Embase) from inception to September 6, 2024. The search strategy was structured into three components and combined as follows:

 #1 PFAS exposure: PFAS, including legacy PFAS, short‐chain PFAS, chlorinated polyfluorinated ether sulfonates (Cl‐PFESA), GenX (HFPO‐DA), ADONA, F‐53B, and other emerging alternatives.
 #2 Thyroid health outcomes: Thyroid function, thyroid‐stimulating hormone (TSH), total/free triiodothyronine (TT3/FT3), andtotal/free thyroxine (TT4/FT4).
 #3 Study population: Pregnant women, pregnancy, fetus, neonate, infant, child, birth cohort, cord blood, and serum.
 Final search: #1 AND #2 AND #3

### 3.2. Inclusion and Exclusion Criteria

The following criteria were employed for the inclusion of articles: (1) subjects were pregnant women; (2) the study design was a cohort study or a cross‐sectional study; (3) the study explored the relationship between short‐chain substitutes for PFAS exposure (at least one of the contaminants) and thyroid health effects (at least one of the indicators, such as TSH, FT3, or FT4); (4) studies must report the essential statistics, such as the level of exposure to PFAS; (5) English article. Exclusion criteria were as follows: (1) off‐topic research; (2) repeated research; (3) only one topic of PFAS exposure or thyroid health effects was reported; (4) literature reviews, conference reports, case reports, letters, and in vivo and in vitro studies.

### 3.3. Study Selection and Screening

**FIGURE 1:**
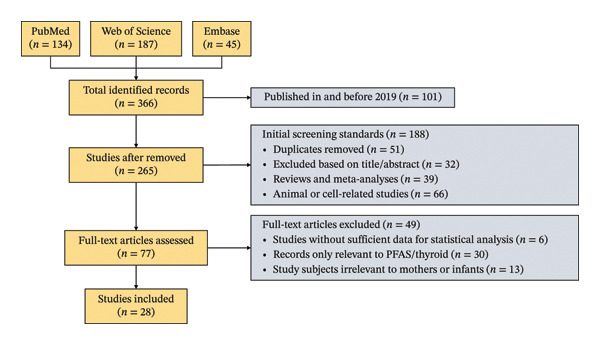
Flowchart of the study search strategy and selection.

A total of 366 records were initially identified across the three databases: 134 from PubMed, 187 from Web of Science, and 45 from Embase. First, we excluded 101 studies published in and before 2019, leaving 265 records for further screening. A two‐step screening process was then implemented in line with the pre‐specified inclusion and exclusion criteria. In the initial title/abstract screening, we removed 51 duplicate records, excluded 32 records deemed irrelevant based on title/abstract, excluded 39 review/meta‐analysis articles, and excluded 66 animal or cell‐related studies, resulting in 188 exclusions at this stage. The remaining 77 records proceeded to full‐text assessment. During full‐text screening, 49 records were excluded: 6 due to insufficient data for statistical analysis, 30 for only focusing on PFAS exposure or thyroid function (not both), and 13 for including study populations irrelevant to mothers or infants. Ultimately, 28 studies were included in this systematic review and meta‐analysis. A detailed PRISMA flow diagram of the study selection process is presented in Figure 1.

### 3.4. Data Extraction and Quality Assessment

We extracted articles from the database to NoteExpress according to the search strategy and automatically removed duplicate items. We first conducted a preliminary screening of the titles and abstracts based on the inclusion and exclusion criteria, then carefully reviewed the full texts and retained those that met the inclusion criteria. Subsequently, we used the Newcastle‐Ottawa Scale (NOS) to independently assess the quality of each study. The selected studies were classified as high (scores ≥ 7), medium (scores = 5–6), or low quality (scores < 5).

## 4. PFAS Exposure in Maternal and Cord Blood

Numerous studies have investigated the levels of PFAS in maternal and infant blood. However, significant variability exists across studies in terms of sample sources, study designs, and reported types of PFAS concentrations, leading to diverse findings and interpretations.

### 4.1. In Maternal Blood

Studies conducted across various countries and regions (Table 2) reveal three main characteristics of PFAS in maternal blood: (1) all types of PFAS are detectable; (2) legacy PFAS remain the dominant compounds; and (3) significant regional differences in exposure levels exist.

**TABLE 2:** PFAS exposure in maternal blood.

Year	Author	Country	Study design	Sample	Sample size	Related PFAS	Concentration (median) ng/mL
*Legacy PFAS*							
2020	Aimuzi et al. [27]	China	Cohort	Maternal plasma	1885	PFOA, PFOS	PFOA: 12.32 PFOS: 9.25

2020	Lebeaux et al. [28]	USA	Cohort	Maternal serum	185	PFOA, PFOS	PFOA: 5.5 PFOS: 14.3

2020	Preston et al. [29]	USA	Cohort	Maternal plasma	726	PFOA, PFOS	PFOA: 5.6 PFOS: 23.9

2020	Chu et al. [30]	China	cohort	Maternal plasma	372	PFOA, PFOS	PFOA: 1.538 PFOS: 7.153

2021	Sarzo et al. [31]	Spain	Cross‐sectional	Maternal plasma	919	PFOA, PFOS	PFOA: 2.55 PFOS: 6.19

2022	Bao et al. [32]	China	Cohort	Maternal serum	50	PFOA, PFOS	PFOA: 27 PFOS: 7.8

2023	Hall et al. [33]	USA	Cohort	Maternal serum	264	PFOA, PFOS	PFOA: 5.6 PFOS: 14

2023	Rivera‐Núñez et al. [34]	USA	Cohort	Maternal serum	285	PFOA, PFOS	PFOA: 0.59 PFOS: 2.5

*PFAS alternatives*							
2020	Aimuzi et al. [27]	China	Cohort	Maternal plasma	1885	PFHxS, PFHpA, PFBS	PFHxS: 0.54 PFHpA: 0.06 PFBS: 0.04

2020	Lebeaux et al. [28]	USA	Cohort	Maternal serum	185	PFHxS	PFHxS: 1.6

2020	Preston et al. [29]	USA	Cohort	Maternal plasma	726	PFHxS	PFHxS: 2.4

2020	Chu et al. [30]	China	Cohort	Maternal plasma	372	Cl‐PFESA	6:2 Cl‐PFESA: 2.405 8:2 Cl‐PFESA: 0.001

2021	Sarzo et al. [31]	Spain	Cross‐sectional	Maternal plasma	919	PFHxS	PFHxS: 0.64

2022	Bao et al. [32]	China	Cohort	Maternal plasma	50	PFHxS, PFHpA, PFBS	PFHxS: 1.3 PFHpA: 7.3 PFBS: 33

2023	Hall et al. [33]	USA	Cohort	Maternal serum	264	PFHxS	PFHxS: 1.5

2023	Rivera‐Núñez et al. [34]	USA	Cohort	Maternal serum	285	PFHxS	PFHxS: 1.72

Regional differences in PFAS concentrations in maternal blood are evident across studies. Median concentrations of legacy PFOA range from 0.59 to 27 ng/mL, and those of PFOS range from 2.5 to 23.9 ng/mL [27–34]. PFOA predominates in Chinese maternal serum, with levels up to 27 ng/mL in Fuxin [32], while PFOS is more dominant in the United States, reaching 23.9 ng/mL in Massachusetts [29]. These differences may reflect industrial patterns, historical usage, and regional environmental exposure pathways. Short‐chain alternatives such as PFHxS are generally present at lower concentrations, ranging from 0.54 to 2.4 ng/mL [27–29, 31, 32], but also vary between China and the United States [27, 29]. Notably, PFBS concentrations reached 33 ng/mL in one Chinese study, indicating the existence of localized contamination hotspots [32].

Overall, legacy PFAS exhibit relatively high exposure levels in maternal blood, with considerable regional variability, which may have a more direct impact on thyroid function. Although concentrations of PFAS substitutes are generally lower, likely owing to their more recent introduction than legacy PFAS, their environmental accumulation trends and localized high‐exposure scenarios underscore the need for continued vigilance.

### 4.2. In Cord Blood

PFAS are widely detected in cord blood, and concentrations are generally lower than those in maternal blood, which reflects the selective barrier function of the placenta [35] (Table 3). Legacy PFAS remain the predominant compounds, though levels vary widely across regions and studies [36–41, 44]. PFAS alternatives, including short‐chain substances and emerging compounds such as 6:2 Cl‐PFESA, are also detectable in cord blood [39, 43, 44]. Notably, alternative PFAS show higher placental transfer efficiency than legacy compounds, suggesting greater potential exposure risks for the fetus [32, 45].

**TABLE 3:** PFAS exposure in cord blood.

Year	Author	Country	Study design	Sample	Sample size	Related PFAS	Concentration (median) ng/mL
*Legacy PFAS*							
2016	Ashley‐Martin et al. [36]	Canada	Cohort	Cord plasma	1301	PFOA, PFOS	PFOA: 0.39 PFOS: 0.15

2016	Fisher et al. [37]	Canada	Cohort	Cord plasma	1385	PFOA, PFOS	PFOA: 0.39 PFOS: b

2018	Richterová et al. [38]	Slovakia	Cohort	Cord serum	322	PFOA, PFOS	PFOA: 0.88 PFOS: 0.64

2020	Liu et al. [39]	China	Cohort	Cord serum	374	PFOA, PFOS	PFOA: 1.65 PFOS: 4.15

2020	Colles et al. [40]	Belgium	Cohort	Cord plasma	269	PFOA, PFOS	PFOA: 1.27 PFOS: 1.11

2021	Guo et al. [41]	China	Cohort	Cord serum	490	PFOA, PFOS	PFOA: 5.6 PFOS: 23.9

2022	Bao et al. [32]	China	Cohort	Cord serum	50	PFOA, PFOS	PFOA: 15 PFOS: 2.4

2023	Costello et al. [42]	China	Cohort	Cord serum	109	PFOA, PFOS	PFOA: 1.457 PFOS: 0.674

2024	Zhou et al. [43]	China	Cohort	Cord serum	789	PFOA, PFOS	PFOA: 4.16 PFOS: 2.66

*PFAS alternatives*							
2016	Ashley‐Martin et al. [36]	Canada	Cohort	Cord plasma	1301	PFHxS,	PFHxS: 0.10

2016	Fisher et al. [37]	Canada	Cohort	Cord plasma	1385	PFHxS,	PFHxS: b

2018	Richterová et al. [38]	Slovakia	Cohort	Cord serum	322	PFHxS,	PFHxS: 0.08

2020	Liu et al. [39]	China	Cohort	Cord serum	374	PFHxS, PFBS Cl‐PFESA	PFHxS: 0.40 PFBS: 0.04 6:2 Cl‐PFESA: 0.78 8:2 Cl‐PFESA: 0.03

2020	Colles et al. [40]	Belgium	Cohort	Cord plasma	269	PFHxS, PFBS	PFHxS: 0.37 PFBS: b

2021	Guo et al. [41]	China	Cohort	Cord serum	490	PFHxS, PFHpA, PFBS	PFHxS: 0.09 PFHpA: 0.21 PFBS: 0.11

2022	Bao et al. [32]	China	Cohort	Cord serum	50	PFHxS, PFHpA, PFBS	PFHxS: 6.8 PFHpA: 16 PFBS: 62

2023	Liu et al. [44]	China	Cohort	Cord serum	109	PFHxS, PFHpA, PFBS Cl‐PFESA	PFHxS: 0.106 PFHpA: 0.094 PFBS: 0.011 6:2 Cl‐PFESA: 0.537 8:2 Cl‐PFESA: 0.025

2024	Zhou et al. [43]	China	Cohort	Cord serum	789	PFHxS, PFHxA, PFBA Cl‐PFESA	PFHxS: 0.92 PFBA: 0.10 PFHxA: b 6:2 Cl‐PFESA: 1.91 8:2 Cl‐PFESA: 0.06

*Note:* b: below limit of detection.

### 4.3. PFAS Exposure in Breast Milk

Breast milk serves as a key postnatal exposure pathway for PFAS in infants [18, 46–50]. Legacy PFAS remain the predominant compounds detected in breast milk, and emerging alternatives including 6:2 Cl‐PFESA have also been widely identified [42, 45] (Table 4). These findings confirm that PFAS alternatives can be transferred from maternal circulation into breast milk, representing a continuous exposure source for breastfed infants.

**TABLE 4:** PFAS exposure in breast milk.

Year	Author	Country	Study design	Sample	Sample size	Related PFAS	Concentration (median) ng/mL
*Legacy PFAS*							
2015	Cariou et al. [46]	France	Cohort	Breast milk	61	PFOA, PFOS	PFOA: b PFOS: b

2020	Jin et al. [47]	China	Cohort	Breast milk	174	PFOA, PFOS	PFOA: 0.033 PFOS: 0.001

2021	Serrano et al. [18]	Spain	Cohort	Breast milk	82	PFOA, PFOS	PFOA: 0.07 PFOS: < 0.00086

2022	Macheka et al. [48]	South Africa	Cohort	Breast milk	50	PFOA, PFOS	PFOA: 0.21 PFOS: 0.05

2023	Yao et al. [49]	China	Cohort	Breast milk	1151	PFOA, PFOS	PFOA: 0.336 PFOS: 0.05

2023	Blomberg et al. [50]	Sweden	Cohort	Breast milk	77	PFOA, PFOS	PFOA: 0.03 PFOS: 0.12

*PFAS alternatives*							
2015	Cariou et al. [46]	France	Cohort	Breast milk	61	PFHxS	PFHxS: b

2020	Jin et al. [47]	China	Cohort	Breast milk	174	PFBA, PFPeA, PFHxA, PFHpA, PFBS, PFHxS, Cl‐PFESA,	PFBA: 0.003 PFPeA: 0.002 PFHxA: b PFHpA: b PFBS: b PFHxS: b 6:2 Cl‐PFESA: 0.016 8:2 Cl‐PFESA: b

2021	Serrano et al. [18]	Spain	Cohort	Breast milk	82	PFHxA, PFHpA, PFBS, PFHxS	PFHxA: 0.0016 PFHpA: 0.0194 PFBS: < 0.0008 PFHxS: < 0.0007

2022	Macheka et al. [48]	South Africa	Cohort	Breast milk	50	PFBA, PFPeA, PFHxA, PFHpA, PFBS, PFHxS	PFBA: 0.05 PFPeA: 0.73 PFHxA: b PFHpA: b PFBS: b PFHxS: b

2023	Yao et al. [49]	China	Cohort	Breast milk	1151	PFBA, PFHxA, PFHpA, HFPO‐DA, PFHxS, Cl‐PFESA,	PFBA: 0.005 PFHxA: b PFHpA: 0.002 HFPO‐DA: b PFHxS: 0.007 6:2 Cl‐PFESA: 0.014 8:2 Cl‐PFESA: b

2023	Blomberg et al. [50]	Sweden	Cohort	Breast milk	77	PFHxS	PFHxS: 0.12

*Note:* b: below limit of detection.

## 5. Effects of Exposure to PFAS on TH Levels in Mothers and Infants

Epidemiological studies across diverse regions have investigated the associations between PFAS exposure and TH levels in mother‒child populations, via statistical methods such as linear regression (Tables 4 and 5).

**TABLE 5:** Effects of exposure to PFAS on TH levels in mothers.

Year	Author	Country	Statistical analysis methods	Effect size
2019	Blomberg et al. [50]	Canada	Mixed effects model	Legacy PFAS‐TH: L‐PFOS‐TSH: 0.005 (−0.031, 0.041) PFAS alternatives‐TH: PFHxS‐TSH: 0.144 (0.039, 0.249); PFHxS‐FT4: −0.006 (−0.012, −0.001)

2020	Aimuzi et al. [27]	China	Linear regression	Legacy PFAS‐TH: PFOA‐TSH: −0.03 (−0.138, 0.078); PFOA‐FT4: 0.121 (0.015, 0.227); PFOA‐FT3: 0.105 (−0.046, 0.256) PFOS‐TSH: −0.016 (−0.099, 0.066); PFOS‐FT4: 0.046 (−0.035, 0.127); PFOS‐FT3: 0.112 (−0.004, 0.227) PFAS alternatives‐TH: PFHpA‐TSH: −0.014 (−0.073, 0.045); PFHpA‐FT4: −0.035 (−0.093, 0.024); PFHpA‐FT3: −0.013 (−0.081, 0.054) PFBS‐TSH: −0.001 (−0.065, 0.063); PFBS‐FT4: 0.016 (−0.048, 0.08); PFBS‐FT3: −0.033 (−0.117, 0.051) PFHxS‐TSH: −0.115 (−0.216, −0.014); PFHxS‐FT4: 0.123 (0.024, 0.222); PFHxS‐FT3: 0.197 (0.054, 0.339);

2020	Lebeaux et al. [28]	USA	Multivariable linear regression	Legacy PFAS‐TH: PFOA‐TSH: 0.09 (−0.14, 0.33); PFOA‐FT4: −0.01 (−0.06, 0.03); PFOA‐FT3: −0.01 (−0.04, 0.01) PFOS‐TSH: 0.02 (−0.24, 0.28); PFOS‐FT4: 0.02 (−0.02, 0.07); PFOS‐FT3: −0.03 (−0.06, 0.00) PFAS alternatives‐TH: PFHxS‐TSH: −0.06 (−0.23, 0.11); PFHxS‐FT4: 0.02 (−0.01, 0.05); PFHxS‐FT3: −0.02 (−0.04, 0.00)

2021	Preston et al. [29]	Spain	Multivariate linear regression	Legacy PFAS‐TH: PFOA‐TSH: 0.0103 (−0.0574, 0.0829); PFOA‐FT4: 0.0242 (−0.0407, 0.0934) PFOS‐TSH: 0.0123 (−0.0584, 0.0883); PFOS‐FT4: 0.0440 (0.0256, 0.119) PFAS alternatives‐TH: PFHxS‐TSH: 0.0609 (−0.0071, 0.134) PFHxS‐FT4: −0.0160 (−0.0756, 0.0475)

2022	Derakhshan et al. [51]	Swedish	Multivariable linear regression	Legacy PFAS‐TH: PFOA‐TSH: 0.06 (−0.01, 0.13); PFOA‐FT4: 0.20 (0.03, 0.36); PFOA‐FT3: 0.04 (−0.001, 0.09) PFOS‐TSH: 0.03 (−0.04, 0.10); PFOA‐FT4: 0.16 (−0.001, 0.33); PFOA‐FT3:0.007 (−0.04, 0.05) PFAS alternatives‐TH: PFHpA‐TSH: 0.003 (−0.06, 0.07); PFHpA‐FT4: 0.23 (0.07, 0.40); PFHpA ‐FT3: −0.01 (−0.06, 0.03) PFHxS‐TSH: 0.01 (−0.05, 0.08); PFHxS‐FT4: 0.13 (−0.01, 0.28); PFHxS‐FT3: 0.01 (−0.03, 0.05)

2022	Jensen et al. [52]	Denmark	Linear regression	Legacy PFAS‐TH: PFOA‐TSH: −1.32 (−7.92, 5.75); PFOA‐FT4: 1.29 (0.21, 2.39) PFOS‐TSH: −1.35 (−8.54, 6.41); PFOS‐FT4: 1.85 (0.66, 3.05) PFAS alternatives‐TH: PFHxS‐TSH: 4.05 (−1.58, 10.00) PFHxS‐FT4: −0.58 (−1.44, 0.28)

### 5.1. In Mothers

Legacy PFAS including PFOA and PFOS are relatively consistently associated with changes in maternal THs, especially FT4 [27, 51, 52] (Table 5). Associations vary slightly across regions but show generally consistent trends. In comparison, PFAS alternatives such as PFHxS, PFBS, and PFHpA show weaker and less stable effects on thyroid function due to lower exposure levels, limited epidemiological evidence, and differences in toxicological profiles compared with legacy PFAS [51].

### 5.2. In Infants

PFAS exposure is also associated with changes in TH levels in infants, although findings are more variable than in mothers [28, 41, 53–55] (Table 6). Legacy PFAS show relatively more consistent associations, while effects of short‐chain and novel alternatives remain weaker and less studied [28, 41]. The inconsistent results may be attributed to differences in sample size, exposure assessment, statistical adjustment, and physiological characteristics across study populations.

**TABLE 6:** Effects of exposure to PFAS on TH levels in infants.

Year	Author	Country	Statistical analysis methods	Effect size
2014	Pande and Anjankar [54]	China	Linear regression	Legacy PFAS‐TH: PFOA‐TSH: −0.498 (−1.464, 0.468); PFOA‐FT4: −0.029 (−0.062, 0.004); PFOA‐FT3: −0.01 (−0.06, 0.03) PFOS‐TSH: −0.083 (−0.292, 0.127); PFOA‐FT4: 0.001 (−0.006, 0.008); PFOA‐FT3: −0.03 (−0.07, 0.02) PFAS alternatives‐TH: PFHxS‐TSH: 0.493 (−1.449, 2.434); PFHxS‐FT4: −0.030 (−0.098, 0.039)

2020	Preston et al. [29]	USA	Multivariable linear regression	Legacy PFAS‐TH: PFOA‐T4: −0.24 (−0.90, 0.42) PFOS‐T4: −0.12 (−0.76, 0.52) PFAS alternatives‐TH: PFHxS‐T4: −0.37 (−0.88, 0.13)

2020	Lebeaux et al. [28]	USA	Multivariable linear regression	Legacy PFAS‐TH: PFOA‐TSH: 0.06 (−0.08, 0.19); PFOA‐FT4: −0.01 (−0.04, 0.03); PFOA‐FT3: −0.01 (−0.06, 0.03) PFOS‐TSH: 0.09 (−0.06, 0.25); PFOA‐FT4: 0.046 (−0.035, 0.127); PFOA‐FT3: −0.03 (−0.07, 0.02) PFAS alternatives‐TH: PFHxS‐TSH: 0.05 (−0.05, 0.16); PFHxS‐FT4: −0.01 (−0.04, 0.02); PFHxS‐FT3: −0.02 (−0.05, 0.02)

2021	Guo et al. [41]	China	Multivariable linear regression	Legacy PFAS‐TH: PFOA‐TSH: −0.074 (−0.171, 0.022); PFOA‐FT4: 0.008 (−0.015, 0.031); PFOA‐FT3: −0.008 (−0.034, 0.018) PFOS‐TSH: −0.012 (−0.156, 0.131); PFOS‐FT4: 0.042 (0.009, 0.076); PFOS‐FT3: 0.013 (−0.024, 0.051) PFAS alternatives‐TH: PFHpA‐TSH: −0.055 (−0.109, −0.001); PFHpA‐FT4: 0.011 (−0.002, 0.024); PFHpA‐FT3: −0.001 (−0.016, 0.013) PFHxS‐TSH: −0.098 (−0.234, 0.027); PFHxS‐FT4: 0.023 (−0.007, 0.052); PFHxS‐FT3: 0.018 (−0.016, 0.051) PFBS‐TSH: −0.068 (−0.140, 0.004); PFBS‐FT4: −0.001 (−0.018, 0.016); PFBS‐FT3: 0.001 (−0.018, 0.020)

2022	Yao et al. [55]	China	Separate multivariable linear regression	PFAS alternatives‐TH: PFBS‐TSH: −1.16 (−1.49, −0.86); PFBS‐FT4: −1.16 (−1.75, −0.63)

2024	Zhang et al. [53]	China	Generalized linear regression	Legacy PFAS‐TH: PFOA‐TSH: 0.05 (−0.05, 0.15); PFOA‐FT4: 0.00 (−0.02, 0.02); PFOA‐FT3: −0.03 (−0.05, −0.00); PFOS‐TSH: 0.05 (−0.07, 0.17); PFOA‐FT4: 0.02 (−0.01, 0.04); PFOA‐FT3: −0.00 (−0.03, 0.03) PFAS alternatives‐TH: PFHpA‐TSH: −0.05 (−0.10, 0.00); PFHpA‐FT4: 0.00 (−0.01, 0.01); PFHpA‐FT3: 0.01 (−0.01, 0.02) PFBS‐TSH: −0.01 (−0.07, 0.05); PFBS‐FT4: 0.01 (−0.00, 0.02); PFBS‐FT3: 0.03 (0.01, 0.04) PFHxS‐TSH: −0.04 (−0.16, 0.07); PFHxS‐FT4: 0.01 (−0.01, 0.04); PFHxS‐FT3: 0.02 (−0.01, 0.05)

## 6. Mixture Effects of Combined PFAS Exposures

PFAS are usually present in complex mixtures, not as simple compounds. These mixtures are found in high concentrations in pregnant women and developing fetuses, and they are very harmful. In a Chinese birth cohort, the median concentration of total PFAS mixtures was 71.91 ng/mL, and combined exposure was significantly inversely associated with maternal FT4 and TSH levels in WQS models [56]. A separate Chinese birth cohort further demonstrated that elevated PFAS mixtures were associated with higher cord plasma T3 and FT3 levels, where PFDA, PFUdA, and PFOA exerted the most prominent individual effects [57]. In the HOME study, PFAS mixtures were associated with altered cord serum metabolite profiles, including the TH metabolite 3‐monoiodothyronine‐4‐O‐sulfate, and 49 metabolic pathways. PFHxS and PFOS dominated the mixture weight [28]. Animal studies confirmed dose‐additive effects of combined PFAS exposure, leading to 20%–30% reductions in maternal THs, elevated neonatal mortality, and long‐term cardiac changes in offspring [58]. Environmentally relevant PFAS mixtures also disrupted maternal metabolism, renal function, TH balance, and placental gene expression in rabbit models, supporting biologically plausible mixture toxicity in humans [59]. To fully characterize real‐world mixture effects, current epidemiological studies widely adopt advanced statistical methods including WQS, BKMR, and quantile‐based g‐computation (QGC), which enable identification of key contributors and interactive effects [28, 56, 57]. Future research on mother–child populations should prioritize these mixture models to quantify cumulative risks, clarify synergistic or additive interactions, and strengthen the translational relevance of thyroid disruption risk assessment.

## 7. Mechanisms of PFAS on Thyroid Function

### 7.1. Mechanisms of Legacy PFAS

Clinically, legacy PFAS disrupt the hypothalamic–pituitary–thyroid (HPT) axis and cause measurable thyroid dysfunction, including abnormal TSH, FT4, and FT3 levels, thyroid tissue injury, and elevated risk of thyroid diseases [11, 20]. Current evidence suggests that PFAS disrupt thyroid function by interfering with TH synthesis, transport, receptor signaling, and gut microbiota (GM) homeostasis.

#### 7.1.1. Interference With TH Synthesis

During TH synthesis, PFAS impair iodine uptake and activation, and disrupt iodination and tyrosine condensation reactions, via multiple mechanisms [60]. Specifically, PFAS alter thyroid peroxidase activity and the expression of type 1 deiodinase, directly or indirectly disrupting TH homeostasis and potentially contributing to hyperthyroidism [60, 61]. Furthermore, PFAS activate nuclear receptors, including peroxisome proliferator‐activated receptor alpha (PPARα), thereby indirectly modulating TH metabolism and synthesis [61]. This pathway is supported by consistent epidemiological findings in pregnant women and children, where PFAS exposure is linked to altered TSH and FT4 levels [27, 62], which align with in vitro and animal evidence of TPO inhibition and deiodinase dysregulation [60, 61].

#### 7.1.2. Disruption of TH Transport

In TH transport, PFAS bind to carrier proteins, such as thyroxine‐binding globulin (TBG), transthyretin (TTR), and albumin (ABL), inducing structural and functional alterations [63–65]. This disruption impairs TH transport, reducing TH availability at target tissues and compromising biological functions. This mechanism is also strongly supported: in vitro competition assays demonstrate PFAS binding to TBG/TTR [63, 64], and human biomonitoring studies link PFAS exposure to reduced TH fractions [66, 67], confirming this pathway in both experimental and epidemiological settings.

#### 7.1.3. Alteration of Receptor Signaling

PFAS also disrupt thyroid receptors and their associated signaling pathways [68, 69]. For instance, PFAS bind to the TSH receptor, disrupting TSH signal transmission, or competitively occupy TH binding sites, thereby altering TH pathways and inducing metabolic imbalances [70]. These disruptions can trigger oxidative stress, immunosuppression, and dysregulation of receptor‐mediated signaling, ultimately causing thyroid dysfunction [68, 70]. Evidence for this pathway is moderate, as most data derive from in vitro and rodent studies [68, 70]; only a few human observational studies link PFAS exposure to TSH receptor autoantibodies or receptor gene expression changes [62], with inconsistent findings.

#### 7.1.4. Disruption of GM Homeostasis

Additionally, PFAS disrupt the TH system by altering GM homeostasis. The GM influences TH levels by modulating iodine uptake, degradation, and enterohepatic circulation [71]. Studies have demonstrated that high PFAS exposure alters the GM composition in pregnant women, suggesting a potential mechanism by which PFAS disrupt TH homeostasis [43, 72].

### 7.2. Mechanisms of PFAS Alternatives

Like legacy PFAS, PFAS alternatives trigger clinical thyroid dysfunction through similar core pathways, but their unique physicochemical properties lead to stronger tissue accumulation, higher placental transfer, and greater thyroid‐disrupting effects in mother–child populations [23, 45].

#### 7.2.1. High Protein‐Binding Affinity and Bioaccumulation

Short‐chain PFAS alternatives exhibit pronounced protein‐binding affinity and persistent bioaccumulation potential. Experimental evidence indicates their preferential accumulation in protein‐ and lipid‐rich tissues [73]. For instance, PFHpA demonstrates prolonged biological retention due to its strong protein‐binding capacity, hindering metabolic clearance [74]. During systemic circulation, PFHpA disrupts thyroid homeostasis by competitively binding to transport proteins or receptors. Evidence for this pathway is experimental‐dominant: in vitro and animal studies confirm strong protein binding and tissue accumulation [73, 74], but human epidemiological data on short‐chain PFAS and TH changes remain limited [20].

#### 7.2.2. High Hydrophilicity and Thyroid Tissue Accumulation

Short‐chain PFAS alternatives are characterized by high hydrophilicity [75]. Owing to its high hydrophilicity, GenX accumulates and distributes in thyroid tissue [76]. This property enhances the accessibility of GenX to thyroid cells, potentially impairing thyroid function. However, further research is needed to elucidate the specific effects of GenX and other short‐chain alternatives on thyroid function.

#### 7.2.3. Disruption of Fatty Acid Metabolism and TC Risk

Emerging evidence suggests that exposure to PFAS alternatives like PFHxA and PFDoA is associated with an increased TC risk, with PFHxA identified as a primary risk factor [77]. The same study linked this exposure to upregulated free fatty acids in patients, suggesting disrupted fatty acid metabolism as a potential mechanism [77]. However, direct experimental validation of the causal pathway from PFAS exposure to metabolic dysregulation and carcinogenesis remains lacking.

#### 7.2.4. High Placental Transfer Efficiency

Furthermore, PFAS alternatives exhibit higher placental transfer rates [32, 45]. This implies that fetuses are more vulnerable to short‐chain PFAS alternatives, potentially threatening fetal thyroid development. This finding is strongly supported by human cord blood studies confirming higher placental transfer of short‐chain alternatives [32, 45] and animal studies demonstrating fetal TH disruption following maternal exposure [78, 79], aligning with epidemiological concerns for early‐life vulnerability.

## 8. Prevention and Control Strategies

To reduce the risks of thyroid dysfunction induced by legacy and alternative PFAS in mother–child populations, comprehensive prevention and control strategies are urgently needed. First, regulatory supervision should be strengthened by restricting the production and application of toxic PFAS alternatives and establishing strict environmental and human biomonitoring safety limits for all PFAS compounds. Second, exposure control is critical: pregnant and lactating women should minimize contact with PFAS‐containing products such as food packaging, waterproof textiles, and stain‐resistant household items, while drinking water purification systems should be promoted in high‐exposure regions to reduce oral intake. Third, biomonitoring programs should be implemented to routinely detect PFAS concentrations in maternal blood, cord blood, and breast milk, enabling early identification of high‐risk populations. Fourth, clinical intervention should be enhanced, including regular thyroid function screening during pregnancy and early infancy to detect abnormal TSH, FT4, and FT3 levels and enable timely medical management. Integrated implementation of these strategies will help protect vulnerable mother–child populations from PFAS‐related thyroid health hazards.

## 9. Conclusion

This paper systematically reviews the effects of legacy PFAS and their alternatives on thyroid function in mother‒child populations. Studies indicate that maternal blood, infant blood, and breast milk levels of legacy PFAS (e.g., PFOA and PFOS) are significantly higher than those of alternatives, while alternatives exhibit higher placental transfer rates and distinct toxicological characteristics. Epidemiological data demonstrate that legacy PFAS and their alternatives are associated with thyroid dysfunction, with the latter showing stronger effects on TH levels and higher links to TC risk.

It should be noted that considerable heterogeneity exists across existing studies regarding the associations between PFAS exposure and thyroid function, which poses challenges for synthesizing consistent conclusions. The primary sources of this inconsistency can be attributed to several key factors: First, differences in gestational sampling timing lead to variable exposure assessments, as PFAS concentrations and TH levels fluctuate dynamically across trimesters, resulting in divergent effect estimates. Second, regional PFAS exposure patterns vary substantially due to differences in industrial emissions, drinking water contamination, and dietary habits, which directly influence baseline exposure levels and observed associations. Third, iodine nutritional status is a critical modifier: iodine deficiency or excess can independently alter thyroid function, potentially masking or amplifying the effects of PFAS exposure. Fourth, analytical methodologies (e.g., detection limits, sample preparation, and PFAS congener quantification) differ across studies, introducing variability in exposure measurement accuracy. Finally, inconsistent confounding factor adjustment (e.g., failure to account for maternal age, BMI, or socioeconomic status) may distort the true associations between PFAS and THs. These heterogeneous factors must be fully considered when interpreting the findings of this review and formulating targeted prevention strategies, as they directly impact the generalizability and clinical relevance of study results.

Although the current research provides some understanding of the relationship between PFAS and TH levels in mothers and children, significant limitations remain. Future research should focus on the following areas:

1. In‐depth study of PFAS alternatives: Current knowledge on the environmental behavior, bioaccumulation potential, and long‐term health effects of PFAS alternatives remains limited. Future research should elucidate their environmental migration and transformation mechanisms, metabolic pathways, and potential health hazards, particularly their long‐term impacts on thyroid function.
2. Optimization of research methods: Existing studies vary in sample sources, research designs, and exposure assessment methods, limiting the comparability of results. Future efforts should prioritize harmonizing research standards, expanding sample sizes, diversifying study populations, and employing precise exposure assessment methods to increase the reliability and consistency of findings.
3. Understanding combined exposures: In real‐world scenarios, mothers and infants are frequently exposed to multiple PFAS and other environmental contaminants simultaneously. While most current studies focus on individual PFAS, future research should explore the mechanisms and interaction effects of combined exposure on maternal and infant thyroid function.
4. Strengthening mechanistic research: Although the mechanisms by which PFAS affect thyroid function have been partially elucidated, many details remain unclear. For instance, the specific molecular mechanisms through which PFAS alternatives disrupt fatty acid metabolism and impair thyroid cell physiology remain poorly understood. Multiomics integrated analyses (e.g., metabolomics‒epigenomics) can elucidate PFAS transgenerational effects on TH balance. In‐depth investigation of these mechanisms will increase our understanding of PFAS effects of PFAS on thyroid function and provide a theoretical foundation for targeted prevention and intervention strategies.
5. Development of prevention and control strategies: Building on existing research, prevention and control strategies for PFAS exposure in mother‒child populations should be prioritized. Enhanced monitoring of PFAS in consumer products is essential to minimize maternal and infant exposure and safeguard health.

**Nomenclature**
PFOA: Perfluorooctanoic acid
L‐PFOS: Linear‐perfluorooctane sulfonate
PFOS: Perfluorooctane sulfonate
PFBA: Perfluorobutanoic acid
PFPeA: Perfluoropentanoic acid
PFHxA: Perfluorohexanoic acid
PFHpA: Perfluoroheptanoic acid
HPFO‐DA/GenX: Hexafluoropropylene oxide dimer acid
ADONA: Ammonium 4,8‐dioxa‐3H‐perfluorononanoate
PFBS: Perfluorobutane sulfonate
PFPeS: Perfluoropentane sulfonic acid
PFHxS: Perfluorohexane sulfonate
FTS: Fluorotelomer sulfonate
6:2 FTS: 6:2 Fluorotelomer sulfonate
Cl‐PFESA: Chlorinated polyfluoroalkyl ether sulfonate
6:2 Cl‐PFESA: 6:2 Chlorinated polyfluoroalkyl ether sulfonate
8:2 Cl‐PFESA: 8:2 Chlorinated polyfluoroalkyl ether sulfonate
TH: Thyroid hormone
TSH: Thyroid‐stimulating hormone
FT4: Free thyroxine
FT3: Free triiodothyronine
T4: Thyroxine
b: Below the limit of detection

## Author Contributions

Lemin Gong conducted the literature search, constructed tables using data from original manuscripts, and was the first writing author. Yuhan Zhou proofread and supported the manuscript.

## Funding

This study was supported by the National Natural Science Foundation of China, 82304103.

## Disclosure

The authors read and approved the final manuscript.

## Conflicts of Interest

The authors declare no conflicts of interest.

## Data Availability

Data sharing is not applicable to this article as no datasets were generated or analyzed during the current study.
